# Stress tolerance enhancement via *SPT15* base editing in *Saccharomyces cerevisiae*

**DOI:** 10.1186/s13068-021-02005-w

**Published:** 2021-07-06

**Authors:** Yanfang Liu, Yuping Lin, Yufeng Guo, Fengli Wu, Yuanyuan Zhang, Xianni Qi, Zhen Wang, Qinhong Wang

**Affiliations:** 1 CAS Key Laboratory of Systems Microbial Biotechnology, Tianjin Institute of Industrial Biotechnology, Chinese Academy of Sciences, Tianjin, China; 2 National Technology Innovation Center of Synthetic Biology, Tianjin, China; 3 University of Chinese Academy of Sciences, Beijing, China

**Keywords:** *Saccharomyces cerevisiae*, Stress tolerance, General transcription factor, Spt15, Base editing, Point mutation

## Abstract

**Background:**

*Saccharomyces cerevisiae* is widely used in traditional brewing and modern fermentation industries to produce biofuels, chemicals and other bioproducts, but challenged by various harsh industrial conditions, such as hyperosmotic, thermal and ethanol stresses. Thus, its stress tolerance enhancement has been attracting broad interests. Recently, CRISPR/Cas-based genome editing technology offers unprecedented tools to explore genetic modifications and performance improvement of *S. cerevisiae*.

**Results:**

Here, we presented that the Target-AID (activation-induced cytidine deaminase) base editor of enabling C-to-T substitutions could be harnessed to generate in situ nucleotide changes on the *S. cerevisiae* genome, thereby introducing protein point mutations in cells. The general transcription factor gene *SPT15* was targeted, and total 36 mutants with diversified stress tolerances were obtained. Among them, the 18 tolerant mutants against hyperosmotic, thermal and ethanol stresses showed more than 1.5-fold increases of fermentation capacities. These mutations were mainly enriched at the N-terminal region and the convex surface of the saddle-shaped structure of Spt15. Comparative transcriptome analysis of three most stress-tolerant (A140G, P169A and R238K) and two most stress-sensitive (S118L and L214V) mutants revealed common and distinctive impacted global transcription reprogramming and transcriptional regulatory hubs in response to stresses, and these five amino acid changes had different effects on the interactions of Spt15 with DNA and other proteins in the RNA Polymerase II transcription machinery according to protein structure alignment analysis.

**Conclusions:**

Taken together, our results demonstrated that the Target-AID base editor provided a powerful tool for targeted in situ mutagenesis in *S. cerevisiae* and more potential targets of Spt15 residues for enhancing yeast stress tolerance.

**Supplementary Information:**

The online version contains supplementary material available at 10.1186/s13068-021-02005-w.

## Background

As one of the oldest domesticated microorganisms, *Saccharomyces cerevisiae* is a widely used workhorse in traditional brewing and modern fermentation industries [1–6]. However, fermentation performance of this yeast is often challenged by harsh industrial conditions, such as various inhibitory stresses from environments, substrates and products [7–9]. Many efforts of exploiting natural and artificial diversity have been made to understand and improve yeast robustness and stress tolerance, and meanwhile revealing pervasive and vital contributions of point mutations (i.e., changes in single nucleotides leading to nonsynonymous amino acid mutations in proteins) in determining yeast phenotypic traits [10–12]. Nevertheless, most of single nucleotide polymorphisms, including protein-coding variants, are present at very low frequencies [12–14], and their phenotypic consequences can be impacted by the genetic background [15, 16]. Thus, a precise and effectively screening tool of in situ introducing point mutation into yeast genome becomes greatly required in order to artificially generate more stress-tolerant strains as well as unravel the mysterious relationship between massive point mutations and yeast phenotypes in different genetic backgrounds.

CRISPR/Cas-based genome editing tools have been extensively developed and applied to genetic modifications of *S. cerevisiae*, including gene deletion and integration as well as transcriptional activation and interference [17–20]. However, most of these tools are used to produce insertions and deletions rather than nucleotide substitutions, and usually require external donor DNAs. Most recently, CRISPR-based cytosine base editors (CBEs) and adenine base editors (ABEs) generating C:G > T:A and A:T > G:C substitutions, respectively, were established without the requirement of external donor DNA and applied in higher eukaryotes for inducing gene inactivation, transcriptional regulation and especially targeted point mutations [21–26]. Nishida et al*.* developed a Target-AID (activation-induced cytidine deaminase) base editor to enable C-to-T substitutions in *S. cerevisiae* and mammalian cells, which was assessed by introducing a stop codon within the open reading frame [27]. Furthermore, the Targeted-AID was employed to semi-randomly mutagenize mammalian and plant endogenous genes in situ, which would not only provide a forward genetic approach to screen for gain-of-function variants associated with different cell phenotypes at base resolution, but also enable greater diversity in directed evolution strategies of protein engineering by creating new substitutions to protein functionality [23, 28]. By contrast, targeted in situ mutagenesis via base editors has been less explored in *S. cerevisiae* so far [29].

Cell robustness and tolerance towards various stresses largely rely on genome-wide global transcriptional regulation of protein-coding genes, which are transcribed by RNA polymerase II (RNA Pol II) [30–32]. RNA Pol II transcription machinery includes RNA pol II and the general transcription factors, such as the TATA-binding protein (TBP, Spt15) in TFIID, TFIIA, TFIIB, TFIIE, TFIIF, and TFIIH (Fig. 3a) [33, 34]. Structural and mutational analyses have shown that Spt15 as well as its interactions with DNA and other components play a pivotal role in regulating promoter specificity in yeast [35–38]. Furthermore, many transcriptional activators, such as the SAGA and mediator complexes, promote transcription by facilitating Spt15 binding to the TATA box [39], while transcriptional repressors, such as Mot1 and negative cofactor 2 (NC2), hamper this interaction (Fig. 3a) [40, 41]. To elicit yeast robustness and stress tolerances by reprogramming gene transcription, Alper et al*.* developed a cellular engineering approach termed “global transcription machinery engineering (gTME)” via PCR (error-prone polymerase chain reaction) mutations, in which *SPT15* is one of the target genes [42]. This approach has been employed to successfully improve tolerances against ethanol [42–44], hyperosmosis [6, 45], oxidative stress [46] and acetic acid [47] in *S. cerevisiae*, ethanol tolerance and production in *Kluyveromyces marxianus* [48], as well as to increase metabolic flux of the isoprenoid pathway in *S. cerevisiae* [49–51] and tune lipophilic properties in *Yarrowia lipolytica* [52]. However, the methods of generating Spt15 mutants in these studies were confined to error-prone PCR, and the screening efficiencies of plasmid-based mutant libraries were relatively low and time-consuming, thus impeding more identifications of beneficial Spt15 mutants.

Therefore, we proposed that the Target-AID base editor could be harnessed to create stress tolerance variations in *S. cerevisiae* by in situ mutagenizing the general transcription factor gene *SPT15*. First, we computationally scanned potential editing sites and constructed individual gRNA for targeting predesigned PAMs in the genome to obtain the different point mutations in the Spt15. Fermentation capacities of these mutations were evaluated at different stress conditions for elucidating the structure–function relationship of Spt15. Next, comparative transcriptome analysis and pairwise protein structure alignment analysis unravelled the distinctive mechanisms of how the mutation influenced global transcription and the interactions with the promoter DNAs and other proteins in RNA Pol II transcription machinery. In all, this study provided a promising and powerful strategy of enabling greater diversity via base editors for altering yeast phenotypes.

## Results

### Effectiveness validation of the Target-AID base editor in *S. cerevisiae*

**Fig. 1:**
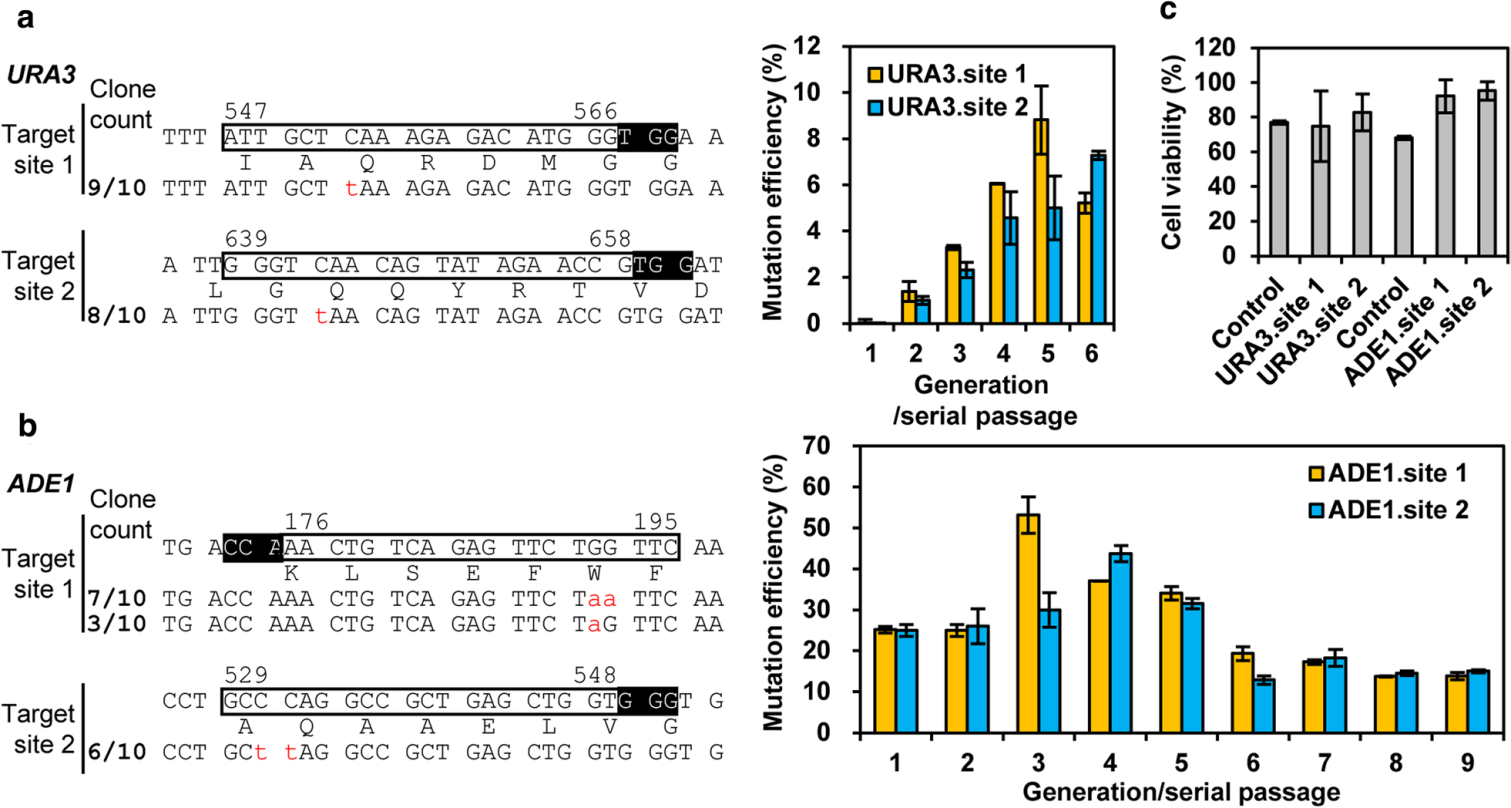
Base editing mediated genomic mutagenesis. **a** Two target sites in *URA3* gene were selected for editing by Target-AID based on the criteria that the cytosine mutations in the − 20 to − 13 position may introduce a stop codon, resulting in a 5-FOA resistance phenotype. **b** Two target sites in *ADE1* gene were selected for introducing a stop codon by Target-AID, resulting in a red colony phenotype. In **a** and **b**, sequences of identified mutations are aligned with the number of colonies over the number of total sequenced colonies for each phenotype change (5-FOA resistance or red colony color). Mutation frequencies of several serial passages are charted on the right. Reference wild-type sequences with translated amino acid sequences are shown with the PAM sequence (inverted) and the target site (box). The target sequence of *ADE1* site 1 is complementary to the sequence shown. **c** Effect of base editing on cell viability. Error bars represent standard deviation among biological triplicates

The Target-AID base editor, composed of gRNA and the fusion protein of Cas9 nickase (nCas9, D10A), PmCDA1 (an AID ortholog) and UGI (uracil DNA glycosylase inhibitor protein), was recently developed to enable C-to-T substitutions without DSB and donor DNA in *S. cerevisiae* [27]. The *CAN1* and *ADE1* genes were targeted to test the effectiveness of Target-AID by introducing a stop codon via C-to-T mutation. Here, to further assess whether the Target-AID would be a generally effective tool to create site-specific point mutagenesis in *S. cerevisiae*, we selected four sites from the *URA3* and *ADE1* genes as targets, following the reported criteria that the cytosine mutations in the − 20 to − 13 position upstream of the PAM may introduce a stop codon (Fig. 1a, b) [27]. The introduction of early stop codons in *URA3* and *ADE1* can lead to their gene disruption and functional loss, thus resulting in a 5-FOA resistance phenotype and a red colony phenotype, respectively. Furthermore, the iterative strategy based on serial passages and commonly used in genome engineering was employed as well [53]. After 5 to 6 generations, the two target sites of *URA3* resulted in approximately 8.8% and 7.3% mutation efficiencies (Fig. 1a). By contrast, the two target sites of *ADE1* resulted in approximately 53.1% and 43.7% mutation efficiencies after 3 to 4 generations (Fig. 1b), which were higher than the previously reported mutation efficiencies of 16% to 47% at other four target sites of *ADE1* [27]. Additionally, base-edited cells showed more than 60% cell viability (Fig. 1c), further confirming that Cas9 nickase-based Target-AID might cause less growth defects or cell death than the full CRISPR/Cas9 nuclease-mediated approaches [19]. These results indicated that gene- and site-specific biases were also observed for Target-AID-mediated point mutagenesis. However, compared to site-specific point mutagenesis by CRISPR/Cas9 practices [54–56], the Target-AID system avoids high cell toxicity and some tedious processes, such as design and usage of heterology block or stuffer to prevent repeated recognition and cutting by the gRNA/Cas9 complex, preparation of donor DNAs, etc. Therefore, Target-AID could provide a powerful and effective tool to generate site-specific point mutagenesis in *S. cerevisiae*.

### Computational scanning and experimental mutagenesis of *SPT15* via Target-AID base editor

**Fig. 2:**
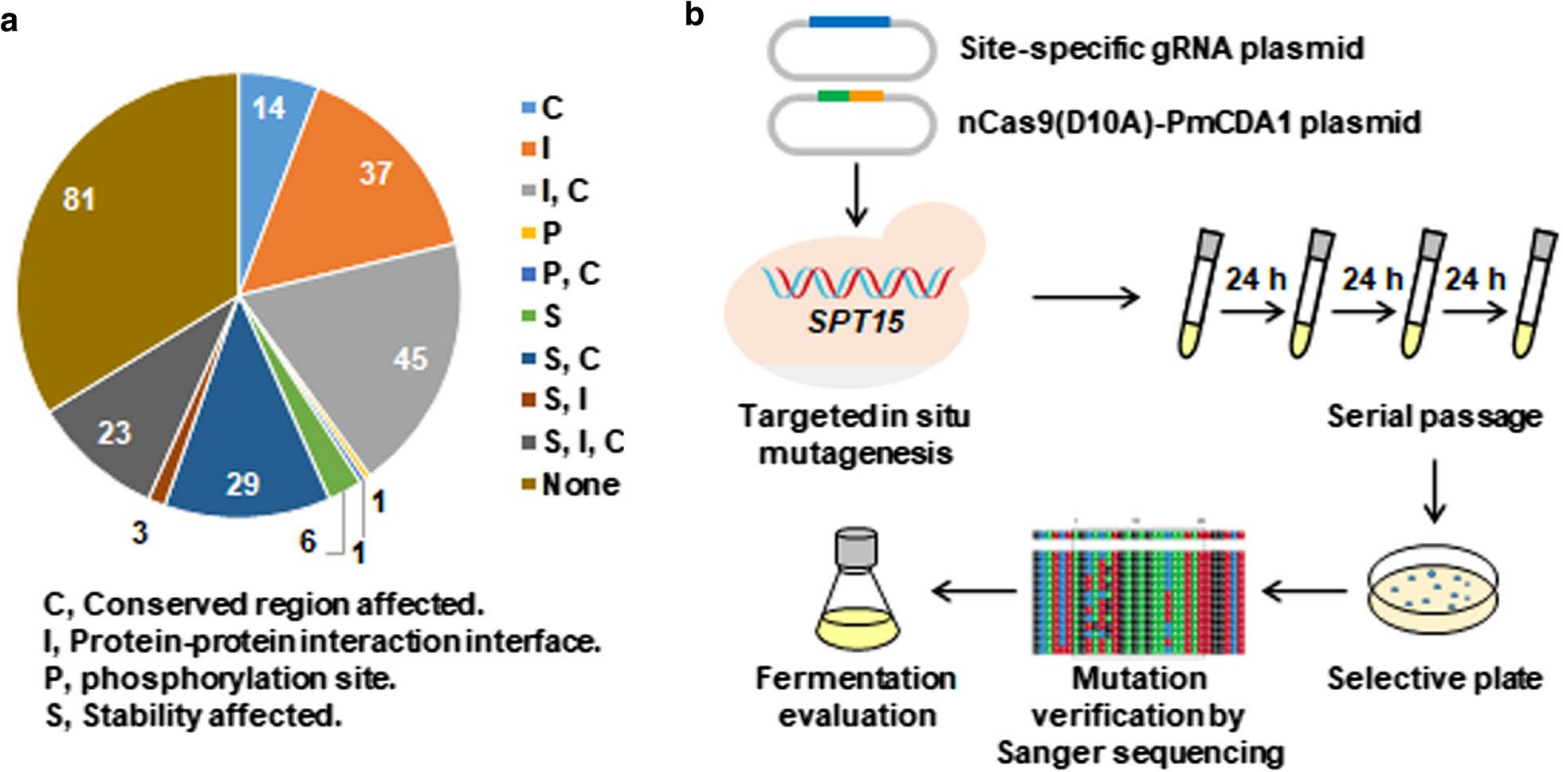
Computational scanning and experimental mutagenesis of *SPT15* via Target-AID base editor. **a** Predicted effects of Spt15 amino acid residues. The predicted functions of amino acid residues were retrieved by artificially applying a point mutation of alanine at each position from the MutFunc database (http://www.mutfunc.com/), which has precomputed data for all possible mutations. Numbers of different variant consequences were counted and labeled on the pie chart. **b** The procedure of in situ mutagenizing Spt15 via base editing as well as stress tolerance genotyping and phenotyping of Spt15 mutant strains

Beyond gene disruption, we aimed to explore the application of the Target-AID in generating nonsynonymous mutations. A general transcription factor Spt15 (TATA-box binding protein), whose point mutations have been partially investigated to influence its interactions with DNA and other proteins in the yeast transcription machinery and/or enhance yeast stress tolerance [35–38], was selected as a target. Spt15 is encoded by a 723-bp nucleotide sequence, and composed of 240 amino acid residues. First, the predicted functions of all the Spt15 amino acid residues were computationally analyzed using the MutFunc database [57]. Approximately two-thirds of 240 amino acid residues were predicted to have single or multiple effects on conserved regions, protein–protein interaction interfaces, phosphorylation, and protein stability (Fig. 2a, Additional file 2: Table S1). Second, the potential Target-AID target sites of the entire Spt15 to introduce nonsynonymous mutations were manually analyzed. These sites should meet two criteria: (i) cytosines localize in the editing window of the − 20 to − 13 position upstream of the PAM on the sense or antisense strands and (ii) codons containing cytosine mutations can lead to nonsynonymous mutations. These potential sites covered 24.6% of total 130 cytosines on the sense strand and 21.4% of total 159 cytosines on the antisense strand (Additional file 3: Table S2), possibly leading to nonsynonymous mutations at 50 positions of the entire Spt15, 60% of which were predicted to have important variant impacts in the MutFunc database (Additional file 2: Table S1). Computational scanning mutagenesis suggested that Spt15 is a feasible and promising target to alter its function by creating nonsynonymous mutations via Target-AID, and thus manipulating yeast stress tolerance.

**Fig. 3:**
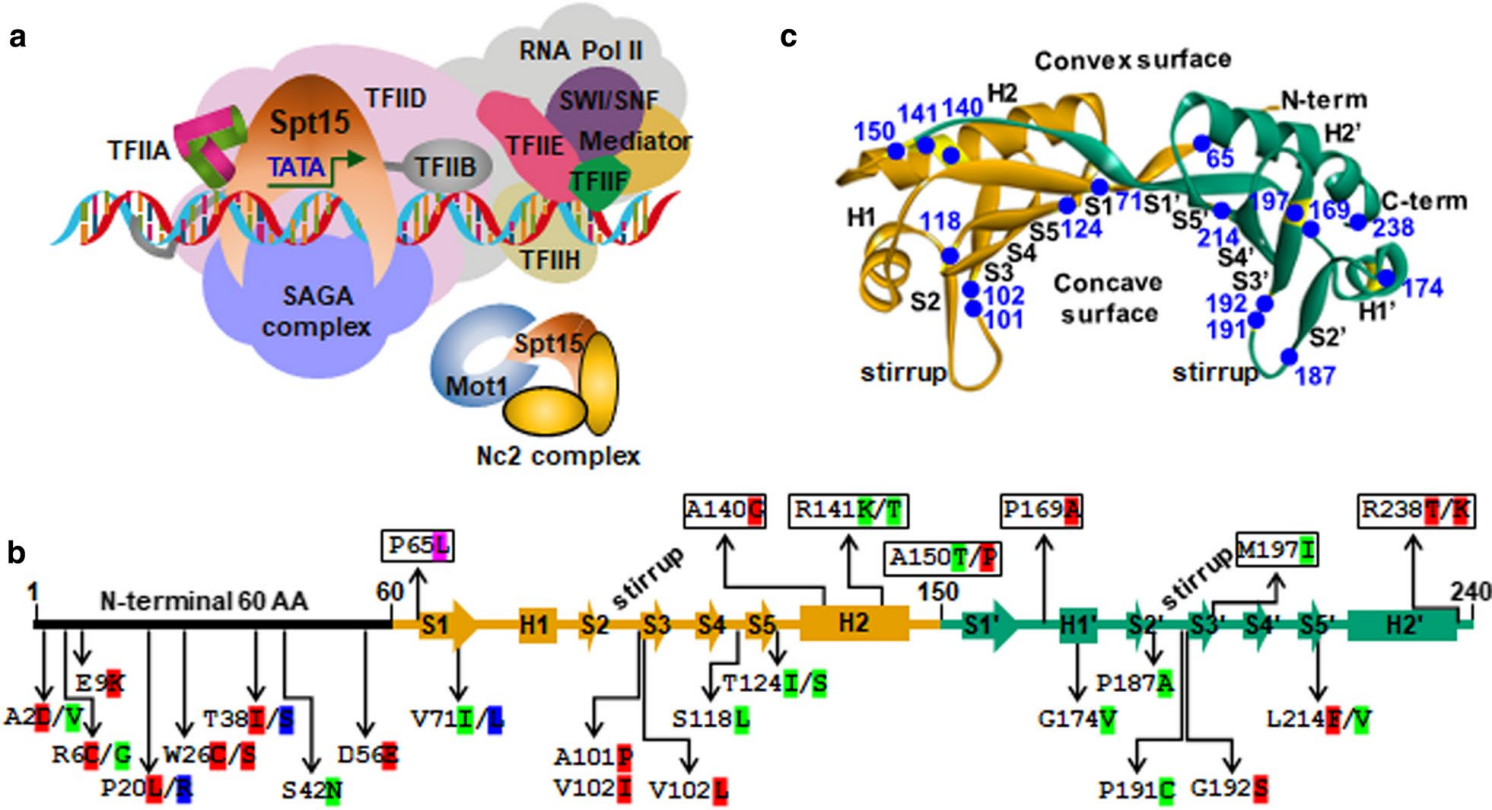
The resulting point mutations of Spt15 and localization. **a** Schematic diagram of interactions among Spt15, DNA and other components in the RNA Pol II general transcription machinery. **b** The resulting 36 Spt15 mutations with altered stress tolerance capacities are mapped on the Spt15 secondary structure. The evaluation results of stress tolerance capacities were extracted from Figs. 4, 5 and Additional file 1: Fig. S1. Common stress-tolerant and sensitive mutations at all three stress conditions (hyperosmotic, thermal and ethanol) are highlighted in red and in green, respectively. Mutations with specific stress-tolerant and opposite stress-resistant capacities at different stress conditions are highlighted in pink and in blue, respectively. P20R and T38S were tolerant to hyperosmotic and thermal stresses, but sensitive to ethanol stress. V71L was tolerant to ethanol stress, but sensitive to hyperosmotic and thermal stresses. P65L was only tolerant to ethanol stress, but showed similar fermentation capacities to the wild-type strain at other conditions. Names of elements of secondary structure follow the convention reported previously [38, 59]. H, represents α-helix. S, represents β-sheet. Mutations localized at the concave surface of the tertiary structure of Spt15 were boxed. **c** Location of the resulting Spt15 mutations on its tertiary structure (PDB 1YTB)

As an experimental practice, individual site-specific gRNA plasmids and the nCas9(D10A)-PmCDA1 plasmid were employed to in situ mutagenize *SPT15* by targeting 33 predesigned PAMs in the genome (Additional file 4: Table S3), and next to screen mutant strains harboring *SPT15* point mutations and with enhanced stress tolerance (Fig. 2b). We obtained total 36 Spt15 mutant strains that had nonsynonymous mutations at half of the 50 predesigned positions of the Spt15 by mutagenizing 49 bases (Fig. 3b). Additionally, except for 28 C-to-T mutations, 19 C to G and 2 C to A mutations were also found (Additional file 3: Table S2). This observation was consistent with the previous report [27], and thus resulting in more than one nonsynonymous mutation at the same amino acid position. Among them, 13 mutants at 8 amino acid positions were localized at the N-terminal region (amino acid residues 1–60). The rest mutants were scattered at 10 β-sheets and 4 α-helixes of the C-terminal domain (residues 61–240), which displays a saddle-shaped tertiary structure as previously reported (Fig. 3b, c) [38]. The concave surface of the saddle is dominated by ten antiparallel β-sheets and responsible for DNA binding, while the convex surface of the saddle is governed by two large and two small α-helixes and outwardly available for interaction with other proteins during transcription initiation [58]. Further mapping of these mutations on the tertiary structure of Spt15 showed that 12 mutants at 9 amino acid positions were localized at the concave surface, and 10 mutants at 7 amino acid positions were localized at the convex surface (Fig. 3c). P187A was situated on the stirrup between sheet S2ʹ and S3ʹ (Fig. 3c). Localization of these mutations in different functional domains implicated distinctive ways in which they might influence the function of Spt15, thus inducing diversified effects on yeast stress tolerance capacities.

### Stress tolerance variations of Spt15 mutations

**Fig. 4:**
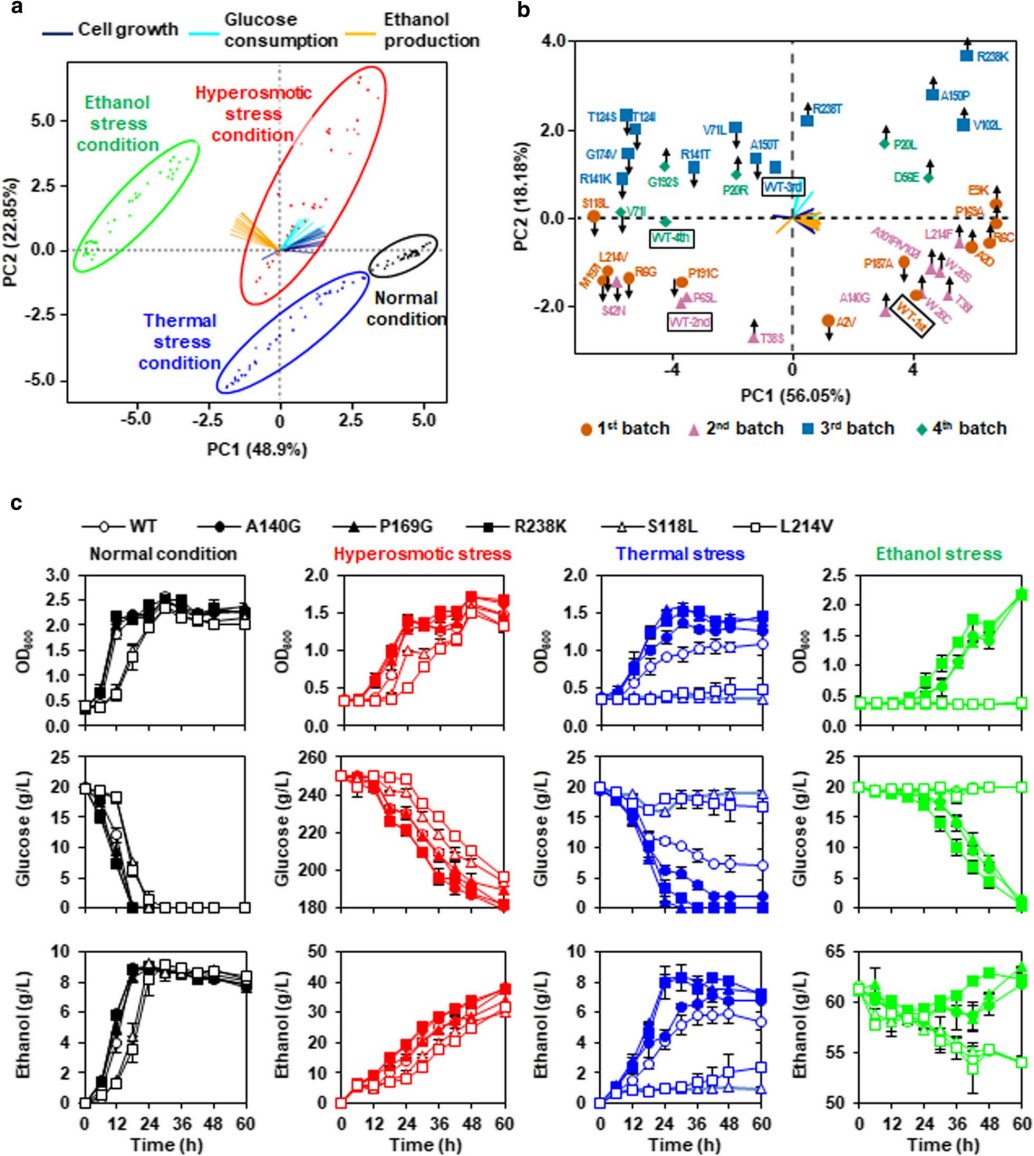
Evaluation of fermentation capacities at normal and three stress conditions. **a** Principal component analysis (PCA) of fermentation data from all the 36 Spt15 mutant strains and the wild-type strain BY4741 at normal and three stress conditions. **b** PCA of fermentation data of the strains at hyperosmotic stress. The strains were evaluated in four batches, indicated using the circle, triangle, square and diamond symbols, respectively. Compared to the wild-type strain in each batch, mutant Spt15 strains showing increased or decreased fermentation capacities are indicated by upward arrow or downward arrow, respectively. In **a** and **b**, fermentation data include cell growth (purple lines), glucose consumption (turquoise lines) and ethanol production (orange lines) during fermentation (hours 0, 6, 12, 18, 24, 30, 36, 42 and 48). Means of biological duplicates are used. **c** Comparison of fermentation performance of three most stress tolerant (A140G, P169G, R238K) and two most stress-sensitive (S118L, L214V) Spt15 mutant strains with the wild-type strain BY4741 at normal, hyperosmotic, thermal and ethanol stress conditions. Error bars represent standard deviation among biological duplicates. In the **c** lower right panel, ethanol level dropped not only during fermentation of the wild-type strain, but also during fermentations of the S118L and L214V strains, which might be because of ethanol evaporation and no cell growth

Fermentation experiments were performed to determine stress tolerance capacities of the Target-AID generated 36 Spt15 mutant strains. First, all the fermentation data (cell growth, glucose consumption and ethanol production) at different time points from all the strains at four conditions including normal, hyperosmotic, thermal and ethanol conditions were subjected to principal component analysis (PCA). The results showed a clear separation of samples into condition-dependent clusters (Fig. 4a). In particular, the variation range of the fermentation data across strains at each of the three stress conditions were much larger than that at normal condition, indicating diversified stress tolerance capacities of these Spt15 mutant strains.

**Fig. 5:**
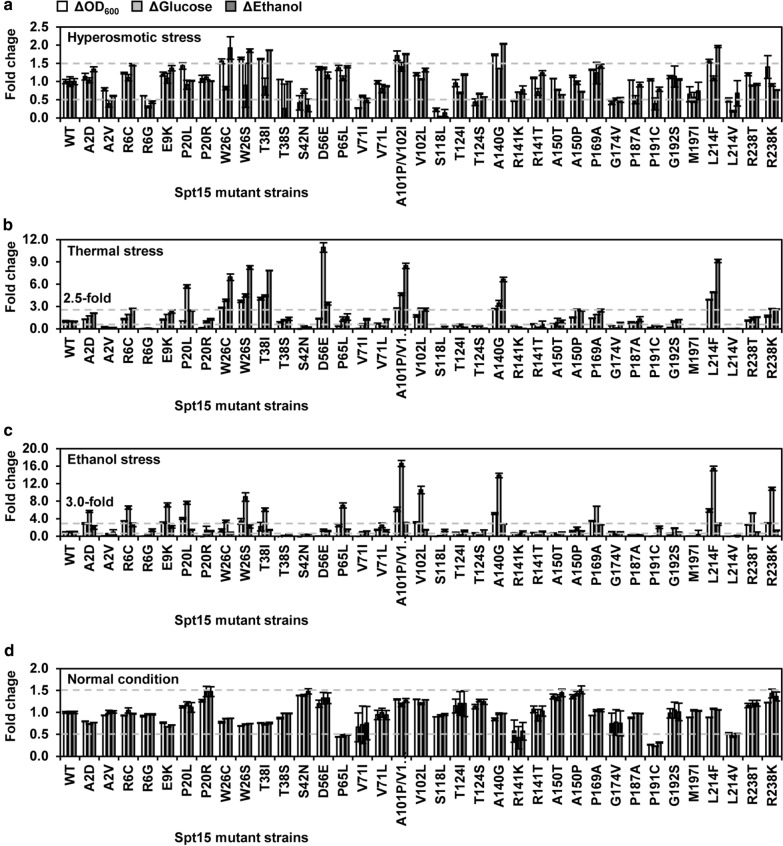
Phenotype comparison of Spt15 mutant and wild-type strains at log phase. Cell growth, glucose consumption and ethanol production were calculated at the 24-h time point at hyperosmotic stress condition **a**, at the 18-h time point at thermal stress condition **b**, at the 30-h time point at ethanol stress condition **c**, and at the 12-h time point at normal condition **d**, respectively. Error bars represent standard deviation among biological duplicates

To further look into the differences of stress tolerance capacities of these Spt15 mutant strains, the fermentation data at each of the three stress conditions were separately used for PCA (Fig. 4b, Additional file 1: Fig. S1a, b). In addition, fold changes of fermentation parameters, such as cell growth (ΔOD_600_), glucose consumption (ΔGlucose) and ethanol production (ΔEthanol), at a certain time point of log phase in comparisons of mutant strains versus the wild-type strain were analyzed as well (Fig. 5). Compared to the wild-type strain at each batch experiment, mutant strains localized at the right or upper area on the PCA plots had increased stress tolerances, while strains situated at the left or lower area had decreased stress tolerances. Since the fermentation capacity and stress tolerance were positively correlated, the farther the mutant strains were separated from the wild-type stain, the more significant the mutant stains were stress tolerant or sensitive. For instance, the mutant strains, such as E9K, P169A and R6C from the first batch, L214F, T38I, W26S and A101P/V102I from the second batch, R238R, A150P and V102L from the third batch, as well as D56E and P20L from the fourth batch, showed relatively higher stress tolerance capacities in contrast to the wild-type strain at hyperosmotic stress condition (Fig. 4b). Additionally, L214F, T38I, W26S and A101P/V102I of the above strains as well as W26C and A140G showed more than 1.5-fold increases of fermentation parameters at the 24-h time point of log phase in contrast to the wild-type strain (Fig. 5a). On the other hand, the mutant strains, such as S118L, M197I, L214V and R6G from the first batch, S42N from the second batch, R141K, G174V, T124S and T124I from the third batch, as well as V71I from the fourth batch, showed relatively lower stress tolerance capacities in contrast to the wild-type strain at hyperosmotic stress condition (Fig. 4b). Additionally, S118L, L214V, R6G, S42N, G174V and V71I of the above strains showed lower than 0.5-fold decreases of fermentation parameters at the 24-h time point of log phase in contrast to the wild-type strain (Fig. 5a).

As for thermal stress condition, the result of PCA distinguished a same set of significantly tolerant and sensitive mutants as hyperosmotic stress condition (Additional file 1: Fig. S1a). Furthermore, L214F, T38I, W26S, A101P/V102I, V102L and D56E of the PCA distinguished stress-tolerant mutants as well as W26C, A140G and R238K showed more than 2.5-fold increases of fermentation parameters at the 18-h time point of log phase in contrast to the wild-type strain, whereas R6G, S42N, S118L, T124I, T124S, R141K, G174V, M197I and L214V of the PCA distinguished stress-sensitive mutants as well as A2V and P191C showed lower than 0.5-fold decreases of fermentation parameters (Fig. 5b).

In terms of ethanol stress condition, the PCA result distinguished a slightly different set of significantly tolerant and sensitive mutants compared to hyperosmotic and thermal stress conditions (Additional file 1: Fig. S1b). To be detailed, the mutant strains, such as P169A, E9K, R6C and A2D from the first batch, A101P/V102I, L214F and A140G from the second batch, V102L, R238K and R238T from the third batch, as well as P20L from the fourth batch, showed relatively higher stress tolerance capacities in contrast to the wild-type strain at ethanol stress condition (Additional file 1: Fig. S1b). Additionally, R6C, E9K, P20L, A101P/V102I, V102L, A140G, P169A, L214F, R238K of the above strains as well as W26S showed more than threefold increases of fermentation parameters at the 30-h time point of log phase in contrast to the wild-type strain (Fig. 5c). On the other hand, the mutant strains, such as L214V, M197I, S118L, A2V, P191C and R6G from the first batch, S42N from the second batch, R141T, R141K, A150T, G174V, T124S and T124I from the third batch, as well as P20R and V71I from the fourth batch, showed relatively lower stress tolerance capacities in contrast to the wild-type strain at ethanol stress condition (Additional file 1: Fig. S1b). Additionally, A2V, R6G, S42N, S118L, P191C, M197I and L214V of the above strains as well as T38S and P187A showed lower than 0.5-fold decreases of fermentation parameters at the 30-h time point of log phase in contrast to the wild-type strain (Fig. 5c).

Besides the above results, the range of the fold changes of fermentation parameters at normal condition was smaller than those at hyperosmotic and thermal conditions (Fig. 5d). Except that P65L, R141K, P191C and L214V showed lower than 0.5-fold decreases of fermentation parameter, the fold changes of fermentation parameters of the rest mutant strains were in a range of 0.5 to 1.5 in contrast to stress conditions (Fig. 5d). This result further confirmed diversified stress tolerance capacities of these Spt15 mutant strains as observed in the PCA result using all the fermentation data (Fig. 4a).

Taken together, whether the 36 Spt15 mutants were stress tolerant or sensitive was qualitatively annotated in Fig. 3b. The number of stress-tolerant mutants obtained at the N-terminal region (amino acid residues 1–60) was eight, whereas the number of stress-sensitive mutants was three. Five stress-tolerant and four sensitive mutants were found to be at the convex surface of the tertiary structure of Spt15. Four stress-tolerant and seven sensitive mutants were found to be at the concave surface of Spt15. Thus, it seemed that the obtained stress-tolerant strains were enriched at the N-terminal region (amino acid residues 1–60) and the convex surface, while the obtained stress-sensitive strains were enriched at the concave surface.

Combining the evaluation results of stress tolerance capacities (Figs. 4, 5, Additional file 1: Fig. S1) and variant impact analysis in the MutFunc database (Additional file 2: Table S1), we selected three stress-tolerant mutant strains (A140G, P169A, R238K) and two stress-sensitive mutant strains (S118L, L214V) at all the three stress conditions for the following transcriptome and protein structural analyses to reveal the underlying mechanisms of regulating yeast stress tolerance by Spt15 mutants. At normal conditions, the A140G, P169A and R238K mutant strains showed similar fermentation performances to the wild-type strain, while the S118L and L214V mutant strains exhibited reduced fermentation performance (Fig. 4c). At stress conditions, the A140G, P169A and R238K mutant strains showed better fermentation performances than the wild-type strain to different extents, while fermentation capacities of the S118L and L214V mutant strains were severely hampered compared to the wild-type strain (Fig. 4c). Thus, these results suggested potentially distinctive regulatory mechanisms underlying different Spt15 mutants.

### Genome-wide reprogramming of global transcription regulated by key Spt15 mutations in response to stress

**Fig. 6:**
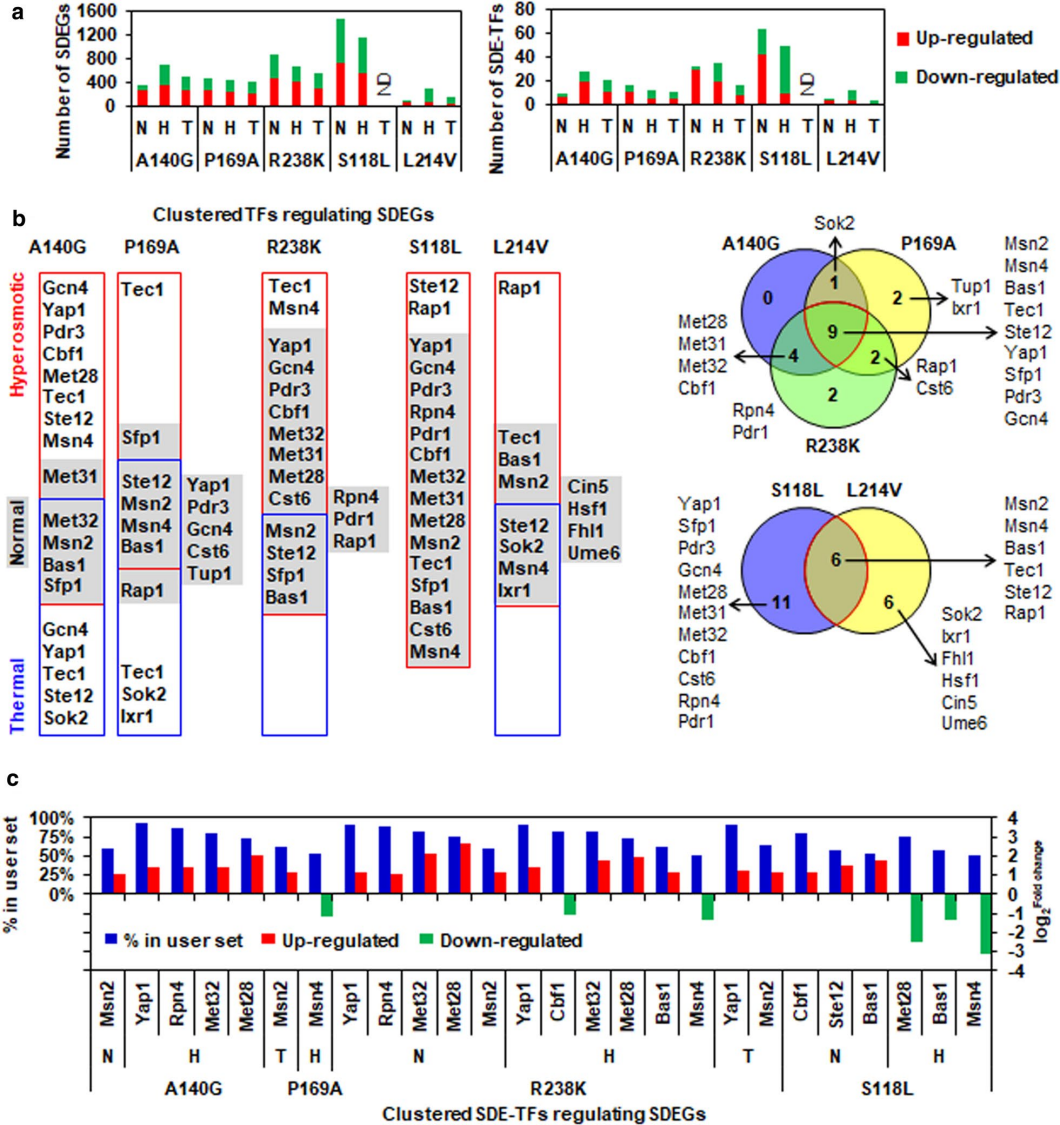
Transcription factor clustering in SDEGs influenced by key Spt15 mutants. **a** Number of SDEGs and SDE-TFs in comparisons of the key Spt15 mutant strains versus the wild-type strain BY4741 at each culture condition including normal (abbreviated as N), hyperosmotic (abbreviated as H) and thermal (abbreviated as T) stress conditions. **b** TFRank-suggested transcription factors clustering in SDEGs and their Venn diagrams among stress-tolerant and sensitive Spt15 mutant strains. **c** TFRank-suggested SDE-TFs clustering in SDEGs. In **b** and **c**, the tool of Rank by TF in the YEASTRACT database was used. Only the percentage of target genes in the user set higher than 50%, which are calculated as the ratio of the number of SDEGs that the TF can regulate to the number of total SDEGs, were considered. *N* normal condition, *H* hyperosmotic stress condition, *T* thermal stress condition, *ND* Not detected

To validate the predicted distinctive regulatory mechanisms underlying different Spt15 mutants (A140G, P169A, R238K, S118L and L214V), genome-wide transcriptome analysis by RNA sequencing was carried out to quantify global transcription changes in the Spt15 mutant strains compared to the wild-type strain at the same culture conditions including the unstressed normal condition as well as hyperosmotic, thermal and ethanol stress conditions. To be noted, RNA sequencing of samples grown at ethanol stress conditions and samples from the S118L mutant strain grown at thermal stress conditions failed due to that qualified RNA cannot be extracted from their severely hampered cell growth. Significantly differentially gene expression (SDEG, absolute fold changes ≥ 2.0 or more; FDR *p*-value ≤ 0.05) in comparisons of the Spt15 mutant strains versus the wild-type strain at each culture condition were extracted, revealing differential global transcription changes in these five Spt15 mutant strains (Additional file 5: Dataset S1, Additional file 6: Dataset S2, Additional file 7: Dataset S3). Regardless of unstressed or stressed conditions, the stress-tolerant strains (A140G, P169A, R238K) displayed hundreds of SDEGs, while the stress-sensitive strain S118L showed more than one thousand SDEGs (Fig. 6a), which seemed to be coincided with its more significant decreased changes of fermentation capacity in contrast to the wild-type stain (Fig. 4c). Although the other stress-sensitive strain L214V showed the least number of SDEGs, the percentage of down-regulated SDEGs was much higher than that of up-regulated SDEGs, which could explain its more significant decreased changes of fermentation capacity in contrast to the wild-type stain (Fig. 4c). Among SDEGs, 4 to 63 significantly differentially expressed transcription factors (SDE-TFs) were found in different mutant strain comparisons and showed similar patterns in terms of the number and the percentages of up- and down-regulated TFs (Fig. 6a).

The Spt15 mutant strains showed quite different alterations of GO biological process enrichment, which were influenced by different culture conditions as well (Additional file 1: Fig. S2, Additional file 8: Dataset S4). It seemed that up-regulated protein translational modification, sulfur assimilation, amino acid biosynthesis and iron ion homeostasis might be beneficial to the acquirement of stress tolerance due to the Spt15 mutations including A140G and P169A. By contrast, the stress-sensitive performance of the S118L strain might be due to the energy imbalance caused by down-regulated ATP-producing carbohydrate metabolism and up-regulated ATP-consuming translation.

As a general transcription factor, Spt15 plays functions by working with many other transcription factors (TFs), such as specific activators and repressors, in preinitiation complex assembly to regulate gene transcription [60]. Amino acid mutations of Spt15 might influence its interplay with specific activators and repressors, thereby altering the global transcription. Thus, we employed the TFRank method in the YEASTRACT database (http://www.yeastract.com) to deduce specific transcription factors and SDE-TFs clustering in SDEGs identified in different Spt15 mutant strains [61, 62]. Clustered TFs and SDE-TFs targeting to more than 50% SDEGs in each comparison were considered (Additional file 9: Dataset S5A, B). Remarkably, most of these clustered TFs were found to be well-known stress-responsive TFs, such as Msn2/Msn4, Bas1, Yap1, Pdr3, etc. (Fig. 6b) [8, 63]. Except for the A140G strain, all the other four mutant strains showed that the great majority of clustered TFs at normal conditions were also enriched at hyperosmotic and thermal conditions (Fig. 6b) [8, 63]. As for the A140G strain, much more TFs were clustered with SDEGs at hyperosmotic and thermal stress conditions than at normal conditions (Fig. 6b). These results suggested that the four Spt15 mutants including P169A, R238K, S118L and L214 might intrinsically interplay with those stress-related TFs at normal conditions, thereby dominantly determining the stress tolerant or sensitive capacities of cells against future stresses. However, the interplay between the A140G mutant and newly involved TFs might be induced by applied stresses, implicating a different role of this amino acid position in the Spt15 from the other four positions.

We further compared the differences of the clustered TFs and SDE-TFs between the stress-tolerant and sensitive Spt15 mutant strains to reveal transcriptional regulatory hubs impacted by the Spt15 mutations. Msn2/Msn4, Bas1, Tec1 and Ste12 were found to be commonly highly clustered (Fig. 6b). Msn2 and Msn4 regulate general stress responsive gene expression against several stresses including heat shock, osmotic shock, oxidative stress, low pH, glucose starvation, sorbic acid and high ethanol concentrations [64, 65]. Msn2/Msn4-regulated genes encode chaperones, certain antioxidant enzymes, housekeeping enzymes, proteases, and other proteins involved in the removal of damaged biomolecules and restoration of metabolic homeostasis or in repair processes [8]. As previously reported, *MSN2* gene expression is constitutive, while *MSN4* expression is Msn2/4 dependent and induced by stress [65]. Here, two factors including Spt15 mutants and environmental conditions should be considered. Msn2, clustering with more than 50% SDEGs that were influenced by the mutant A140G and by R238K at normal non-stressed and thermal stress conditions, showed significantly increased transcription expressions compared to the wild-type strain (Fig. 6c). By contrast, Msn4, clustering with more than 50% SDEGs that were influenced by the mutant P169A, R238K and S118L at hyperosmotic stress conditions, showed significantly decreased transcription expressions compared to the wild-type strain (Fig. 6c). These results suggested that the regulatory effects of Spt15 mutations on Msn2 and Msn4 transcriptions seemed to be specific to normal non-stressed and thermal stress conditions or hyperosmotic stress conditions, respectively. Bas1, regulating some genes in the purine and histidine biosynthesis pathways and in meiotic recombination, is predicted to regulate stress responses under oxidative stresses, temperature shift stresses and osmotic stresses [66]. Bas1 showed significantly increased transcription in the stress-tolerant R238K mutant strain but significantly decreased transcriptions in the stress-sensitive S118L mutant strain grown at hyperosmotic stress conditions. Tec1 and Ste12 are involved in hyperosmotic stress response and regulate most genes in the filamentation/invasion pathway [67]. Tec1 had no significant transcriptional change, whereas Ste12 showed significantly increased transcription in the S118L strain at normal condition.

Yap1, Sfp1, Pdr3 and Gcn4, which are well-characterized TFs in response to various stresses [64, 65], were commonly highly clustered in regulating SDEGs of all three stress-tolerant mutant strains and the stress-sensitive S118L strain, but not the other stress-sensitive L214V strain (Fig. 6b). Additionally, Yap1 showed significantly increased transcription in the A140G strain at normal condition as well as in the R238K strain at normal non-stressed, hyperosmotic and thermal stress conditions compared to the wild-type strain, but not in the P169A and S118L strains. And no significant transcriptional changes were observed for Sfp1, Pdr3 and Gcn4. Most interestingly, Met28, Met31, Met32 and Cbf1, which are cofactors of the Met4 transcription factor controlling the expression of sulfur metabolism and oxidative stress response genes [68, 69], were commonly highly clustered in regulating SDEGs of the stress-tolerant A140G and R238K strains as well as the stress-sensitive S118L strain (Fig. 6b). In the comparisons of the A140G and R238K strains versus the wild-type strain, Met28 and Met32 showed significantly increased transcription at normal and/or hyperosmotic stress conditions, while Met28 showed significantly decreased transcription at hyperosmotic stress condition in the S118L strain. Additionally, Cbf1 showed significantly decreased transcription in the R238K strain at hyperosmotic stress condition, but significantly increased transcription in the S118L strain at normal condition. Besides the above highly commonly clustered TFs, Rap1, Sok2, Rpn4, Pdr1, Cst6 and Ixr1, which are involved in stress response [8, 63, 70, 71], were also observed to be clustered in regulating SDEGs of both the stress-tolerant and sensitive strains, although they had no significant transcriptional changes (Fig. 6b, c). In addition, four other well-known stress-related TFs including Fhl1, Hsf1, Cin5, and Ume6 were specifically clustered in SDEGs of the L214V strain (Fig. 6b) [31, 70, 72].

### Predicted impact of key amino acid changes on protein structure and function of Spt15

**Fig. 7:**
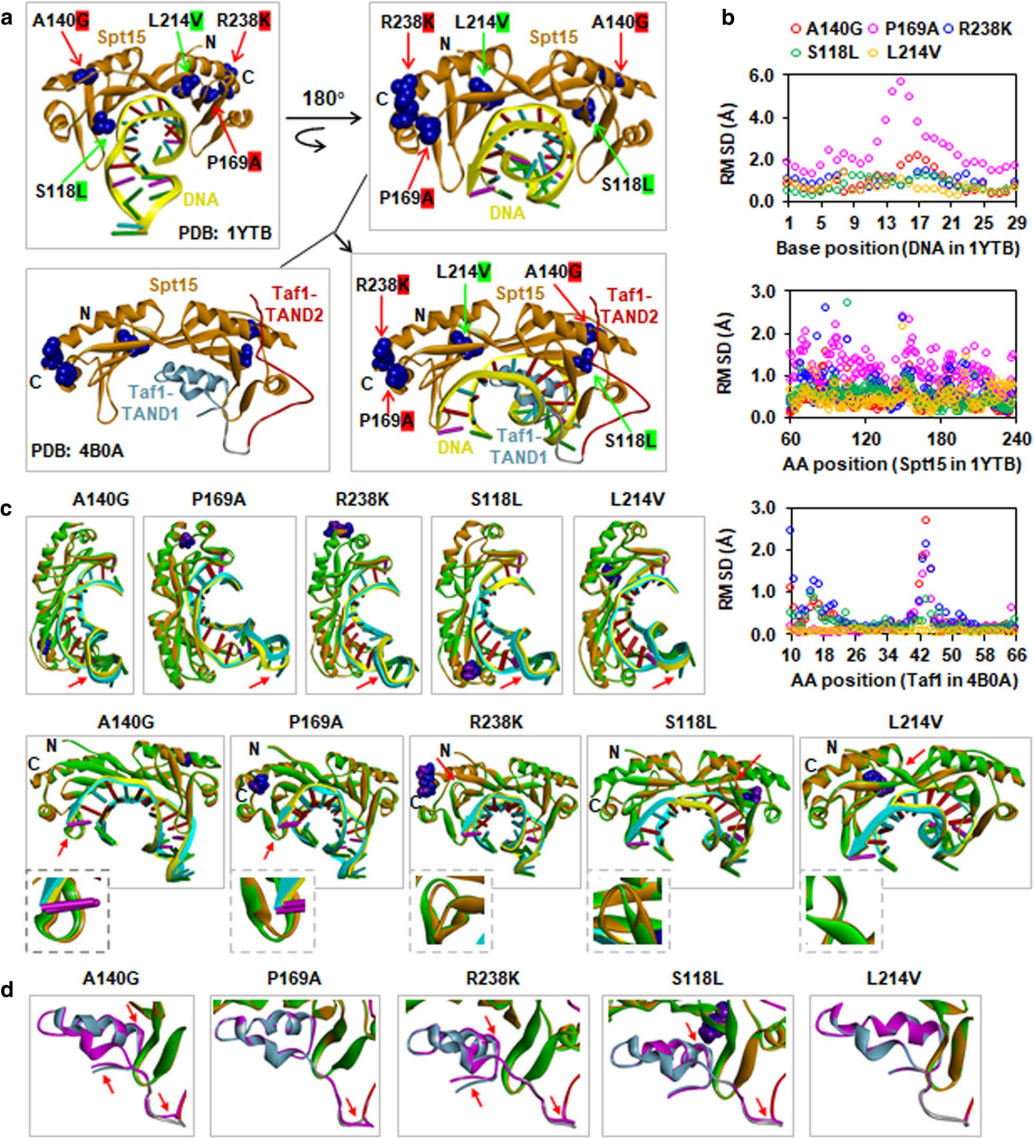
Predicted effects of key point mutations on protein conformation of Spt15 and its interactions with DNA and Taf1. **a** Localization of key point mutation of Spt15 on PDB structures 1YTB and 4B0A including three most stress tolerant (A140G, P169G, R238K) and two most stress-sensitive (S118L, L214V) mutations. Superposition structure of the Spt15-DNA (Chain A and C) from the structure 1YTB and Spt15-Taf1 from the structure 4B0A was constructed using super-alignment in the PyMol program. **b** RMSD (root mean square deviation) profiles of DNA and Spt15 in 1YTB and Taf1 in 4B0A influenced by key Spt15 mutants. **c** DNA and Spt15 conformation changes in 1YTB. **d** Taf1 conformation changes in 4B0A. Conformation changes are indicated by red arrows. The coloring of chains and residues is as follows. Wild-type and mutant Spt15 were shown in orange and green, respectively. DNA interacting with wild-type and mutant Spt15 are in yellow and cyan, respectively. In Taf1 interacting with wild-type Spt15, the TAND1 and TAND2 domains are shown in sky blue and red, respectively. Taf1 interacting with mutant Spt15 is shown in purple. Wild-type and mutant amino acids were shown in blue and purple, respectively

As a general transcription factor, Spt15 binds to the TATA-box and interacts with other factors to form the preinitiation complex at promoters. We predicted that key amino acid changes might have impacts on the interactions of Spt15 with DNA and other proteins by influencing Spt15 conformation, and thereby resulting in genome-wide reprogramming of global transcription in the Spt15 mutant strains. Thus, to assess potential conformational changes caused by amino acid changes, we performed pairwise protein structure alignment between Spt15 point mutation and the wild-type Spt15 in two crystal structures of Spt15-related complexes (PDB 1YTB and 4B0A) (Fig. 7) and calculate root mean square deviation (RMSD) values. 1YTB shows the interaction between Spt15 and the TATA-box DNA [73], whereas 4B0A contains the Spt15 and Taf1 N-terminal domains TAND1 and TAND2 to show competitive multiprotein TBP interplay patterns [74]. Superposition structure of the Spt15-DNA (Chain A and C) from the structure 1YTB and Spt15-Taf1 from the structure 4B0A showed a competitive binding of Taf1 at the concave DNA binding surface (Fig. 7a). The profiles of RMSD versus each position at DNA in 1YTB indicated apparent peaks at positions 11 to 22 of DNA due to the A140G and P169A mutations (Fig. 7b). Correspondingly, significant conformational changes at positions 11 to 22 of DNA, which is localized downstream of the TATA box, were observed in pairwise protein structure alignment between Spt15 point mutations of A140G and P169A and the wild-type Spt15 in crystal structure 1YTB, which were relatively smaller in R238K, S118L and L214V mutants (Fig. 7c). Upon DNA opening during transcription initiation, melting begins about 20 base pairs downstream of the TATA box [58, 75]. Thus, Spt15 point mutations of A140G and P169A might impact DNA opening during transcription initiation. The RMSD profiles at amino acid positions of 60 to 240 in Spt15 of 1YTB implicated that these five mutations might have effects on Spt15 conformational changes to different extents (Fig. 7b). The P169A mutation seemed to induce a bigger conformational change than the A140G mutation at the C-terminal stirrup (Fig. 7c). The R238K mutation caused an obvious conformational change between the sheets S3ʹ and S4ʹ. The S118L mutation at the end of the S4 sheet resulted in an apparent conformational change between the sheets S3 and S4. The L214V mutation induced a slight conformational change between the S5ʹ sheet and the H2ʹ helix. These differential conformational changes of Spt15 suggested distinctive impacts of these five mutations on the function of Spt15.

The profiles of RMSD versus each position at 10 to 66 of Taf1 in 4B0A showed small peaks at 10 to 24 positions and sharp peaks at 40 to 48 positions due to the A140G, R238K and S118L mutations, respectively. As for the P169A mutation, a sharp peak at 40 to 48 positions was also observed (Fig. 7b). Correspondingly, conformational changes at the N-terminus of Taf1-TAND1 were found due to the A140G, R238K and S118L mutations (Fig. 7d). Conformational changes between the Taf1-TAND1 and Taf1-TAND2 regions were observed due to the A140G, P169A, R238K and S118L mutations (Fig. 7d). But for the L214V mutation, no apparent RMSD peak and conformation changes in Taf1 were found (Fig. 7b, d). Remarkably, the A140G mutation seemed to induce the most significant conformational changes of Taf1, implicating that the A140G mutation might have the most significant potential impact on competitive multiprotein TBP interplay patterns [74].

## Discussion

CRISPR-mediated base editors have been employed for genetic modifications to perturb cell phenotypes by producing gene inactivation [76–78]. Besides, most recently, the Target-AID base editor was used to introduce both gene inactivation and site-specific point mutations at the genome scale to identify key amino acid residues with significant impacts on *S. cerevisiae* fitness [29]. Here, we demonstrated for the first time that the Target-AID can be used for semi-precisely protein engineering of yeast to enhance its stress tolerance capacities, in which the general transcription factor gene *SPT15* was in situ mutagenized for reprogramming global gene transcription. In previous studies, the gTME technique targeting *SPT15* has successfully generated and screened dozens of Spt15 point mutations to improve several stress tolerances *of S. cerevisiae* [6, 42–47]. Compared with the mutation generation and screening approaches of error-prone PCR and plasmid-based mutant libraries in the gTME, the method of using individual site-specific gRNA plasmid showed apparent advantages of producing more predesigned mutations and higher effectiveness. Remarkably, the Targeted-AID allowed in situ mutagenesis, which could ensure the consistent functional consequences of changes in the native context. Therefore, this study manifested that the base editors would be a precise and feasible tool for in situ protein engineering and phenotype enhancement of microorganisms.

Spt15 is a key player in RNA Pol II transcription machinery regulating genome-wide gene transcription by binding the TATA box of gene promoters and interacting with other transcriptional regulators [34]. Many structural and mutational investigations have revealed key residues influencing the above interactions [35–38]. Furthermore, it has been reported that some point mutations of Spt15 influenced different sets of gene expression according to transcriptome analysis of ethanol or acetic acid stress-tolerant Spt15 mutants previously generated by the gTME [42, 43, 47]. In this study, comparative transcriptome analysis of three most stress-tolerant (A140G, P169A and R238K) and two most stress-sensitive (S118L and L214V) mutants confirmed that different point mutations of Spt15 induced some common and distinctive global transcription reprogramming and transcriptional regulatory hubs in response to stresses (Fig. 6). Thus, it was interesting to know the structural impacts of a single mutation in Spt15 behind its regulatory function. Pairwise protein structure alignment between Spt15 point mutation and the wild-type Spt15 in two crystal structures of Spt15-related complexes (PDB 1YTB and 4B0A), which display the interactions of Spt15 with the TATA-box DNA [73] and Taf1 N-terminal domains TAND1 and TAND2 to show competitive multiprotein TBP interplay patterns [74], respectively, provided some insights (Fig. 7).

Two most stress-tolerant Spt15 mutations including A140G and P169A seemed to apparently facilitate the DNA opening of about 20 base pairs downstream of the TATA box during transcription initiation due to their significant impacts on the conformational changes of DNA in PDB 1YTB (Fig. 7), although they are localized at the convex surface of the saddle-shaped structure of Spt15 and have no direct interaction with DNA (Fig. 3c). Remarkably, the A140G mutation induced significant conformational changes of Taf1 in PDB 4B0A (Fig. 7), suggesting their potential impacts on competitive multiprotein TBP interplay patterns because of highly similar TBP-binding motifs also presented by the structurally distinct TFIIA, Mot1 and Brf1 proteins [74]. Additionally, the interactions of the A140R and R238D mutations with Brf1 were reported to show biochemical defect [79], suggesting the participations of the residues at 140 and 238 positions in interacting with Brf1, maybe also TFIIA and Mot1 [80]. Mot1–Spt15 complex was previously reported to be an inactive form under normal growth condition, but become active in response to environmental stresses [81]. Thus, the A140G mutation might also influence its interaction with Mot1, which could explain why the A140G mutation influenced more differential gene expression and induced more clustered TFs under stress conditions than those under normal non-stress condition in contrast to other mutations (Fig. 6).

By contrast, the effect of the R238K mutation on the activity of Mot1–Spt15 complex in response to stresses seemed to be unclear. On the other hand, the two most stress-sensitive S118L and L214V mutations, which are localized at the concave surface of the Spt15 structure, showed direct interaction with DNA (Fig. 7a). Furthermore, the S118L mutation has been reported to be defective in DNA binding [79], which could explain its most significantly disturbed transcriptome compared to other mutations. Most interestingly, the N-terminal region (amino acid residues 1–60), the removal of which enhances TBP-TATA association and TBP dimerization in vitro, but rescues the transcriptional defects of a subset of mutations that disrupt DNA binding in vivo [79], showed an enriched stress-tolerant mutations (Fig. 3b). Since Spt15 plays a central function in regulating global transcription, it would be intriguing to further construct and investigate more Spt15 mutations based on our insights into the impacts of the total obtained 36 Spt15 mutations on yeast stress tolerances.

In this study, we employed the Targeted-AID as an example to prove that CRISPR/Cas-mediated base editing technology would be a powerful and feasible tool for in situ protein engineering and phenotype enhancement of microorganisms. Mostly recently reported dual cytosine and adenine base editors [28, 82] as well as Cas9 variants with different PAM sequences [83] could further expand the spectrum of predesigned base editing targets. In addition, our study further emphasized Spt15 is a promising target for engineering to promote complexed phenotypes of *S. cerevisiae*, such as stress tolerance.

## Conclusions

Creating artificial phenotypic variations of yeast is a critical approach to promote its applications. In this work, we harnessed the Target-AID base editor of enabling C-to-T substitutions for targeted in situ mutagenesis of a general transcription factor gene *SPT15* in *S. cerevisiae* and greatly altered yeast stress tolerance capacities. N-terminal and convex localized amino acid changes of Spt15 favored yeast stress tolerance. Different key Spt15 mutants controlled distinctive transcription reprogramming and regulatory hubs, which might be due to the changed interactions of Spt15 mutants with DNA and other proteins in RNA Polymerase II transcription machinery. These results demonstrated that Target-AID base editor could be an efficient tool to advance in situ mutagenesis of yeast genes, and thus enhancing its phenotypes. Furthermore, this study provided new insights into the impacts of Spt15 mutants on global transcription reprogramming and yeast stress tolerance.

## Methods

### Yeast strains and culture conditions

*Saccharomyces cerevisiae* strains BY4741 (*MATa; his3Δ1; leu2Δ0; met15Δ0; ura3Δ0*) (EUROSCARF, Oberursel, Germany) and BY4741a (*MATa; his3Δ1; leu2Δ0; lys2Δ0; met15Δ0; ura3Δ0; pdc1::ura3*) [84] were used as hosts. Yeast strains were cultured in the Synthetic Dropout (SD) medium minus the auxotrophic amino acids (FunGenome, Beijing, China) at 30 °C. When inducing base editing, yeast cells containing base editing plasmids were cultured in SD medium with 20 g/L galactose and 10 g/L raffinose. For hyperosmotic stress tolerance phenotyping, SD medium containing 250 g/L glucose was used. For thermal stress tolerance phenotyping, experiments using SD medium with 20 g/L glucose were performed at 40 °C. For ethanol stress tolerance phenotyping, SD medium containing 20 g/L glucose and 8% (v/v) ethanol was used. As for normal condition as an unstressed control, experiments were performed at 30 °C using SD medium containing 20 g/L glucose.

### gRNA design and plasmid construction

All potential gRNA sequences targeting to the open reading frames of *URA3*, *ADE1* and *SPT15* genes were designed using the ATUM gRNA design tool [85]. As Nishida et al*.* reported [27], the Target-AID has a various activity at positions from − 20 to − 13 relative to target PAM sequence. Thus, cytidines in this editing window were targeted and further analyzed for their possibilities of introducing stop codons, synonymous or nonsynonymous mutations. For *URA3* and *ADE1* genes, the codons including CGA (Arg), CAG (Gln), and CAA (Gln) in the coding strand as well as CCA in the antisense strand corresponding to GGT (Gly) in the editing window, which create stop codons of TGA (opal), TAG (amber), and TAA (ochre) stop codons by changing the C to T via the Target-AID, were focused to design gRNAs. For the *SPT15* gene, the cytidine-containing codons in the editing window only to create nonsynonymous mutations were focused to design gRNAs.

The cytidine deaminase and Cas9 nickase plasmid pRS315e_pGal-nCas9(D10A)-PmCDA1, which expresses nCas9(D10A)-PmCDA1 under the galactose-induced *GAL1* promoter in yeast cells [27], was purchased from the Addgene distributor Beijing Zhongyuan Ltd. (#79617; Addgene, Cambridge, MA, USA). The gRNA expression cassette consisting of the *SNR52* promoter, the 20-bp guide sequence (complementary region for specific DNA binding) and the *SNR52* terminator was PCR amplified from the pCAS plasmid [86] (#60847; Addgene, Cambridge, MA, USA) by introducing restriction sites of *Xma* I and *BamH* I in the primers (Additional file 4: Table S3), and inserted into the plasmid pRS423 using the restriction cloning method. The resulting plasmid was named pRS423-gRNA. The target gRNA sequences introduced in the primers (Additional file 4: Table S3) were replaced in pRS423-gRNA using the ClonExpress^®^ II One Step Cloning Kit (Vazyme Biotech Co., Ltd, China). To facilitate rapid construction of total 33 individual gRNA plasmids for targeting *SPT15*, all the 33 pairs of specific forward and reverse primers (Additional file 4: Table S3) synthesized and pooled in equal mole amounts by GENEWIZ, Inc. (Suzhou, China) were used for plasmid cloning. Total 22 sequenced unique plasmids were extracted from 96 colonies. The rest 11 individual plasmids for *SPT15* were further separately constructed. All the gRNA plasmids were sequenced by Beijing Tsingke Biotechnology Co., Ltd. (Beijing, China) to verify the insertion of 20-bp sequence.

### Targeted in situ mutagenesis using base editing

One microgram each of the cytidine deaminase and Cas9 nickase plasmid and the gRNA plasmid DNA were co-transformed into *S. cerevisiae* BY4741a used for targeting *URA3* or BY4741 used for targeting *ADE1* and *SPT15* by following the instruction of the Zymo Research Frozen-EZ Yeast Transformation II Kit™ (Zymo Research Corp., Irvine, CA, USA). The positive transformants were selected on SD medium plates without histidine and leucine (SD-His-Leu). Galactose induction of nCas9(D10A)-PmCDA1 expression and base editing was conducted as previously reported [19, 27]. Mutation efficiencies and cell viabilities were calculated as well. Cell viability was calculated by dividing the number of colonies on galactose and raffinose plates by the number of colonies on glucose plates [19]. For mutagenizing *SPT15*, cells after four iterative cycles of inducing base editing were spread on SD-His-Leu plus glucose plates. To verify the editing events, the target regions were PCR amplified from ten colonies and sequenced by the Beijing Genomics Institute (BGI) (Shenzhen, China).

### Stress tolerance phenotyping

The colonies with a unique and sequence-verified *SPT15* nonsynonymous mutation were determined for their stress tolerance properties using flask fermentation at normal, hyperosmotic, thermal and ethanol stress conditions. Fermentation experimental setups, measurements of OD_600_, glucose and ethanol concentrations, as well as principal component analysis (PCA) for fermentation data were performed as previously described [87]. Cells were pre-cultured in SD plus 20 g/L glucose media at 30 °C overnight before applying to fermentation experiments. The initial OD_600_ of 0.4 was used. Fermentation experiments were performed in 50-mL flasks containing 20 mL media under aerobic conditions with shaking at 250 rpm. Optical density (OD) at 600 nm was determined using a platereader (Molecular Devices SpectraMax M2e, San Jose, CA, USA). Concentrations of glucose and ethanol were measured by high-performance liquid chromatography (HPLC) on an Agilent 1260 system (Agilent, Santa Clara, CA, USA) equipped with a refractive index detector and a Phenomenex RFQ fast acid column (100 mm × 7.8 mm ID) (Phenomenex Inc., Torrance, CA, USA). The column was eluted with 0.005 mol/L H_2_SO_4_ at a flow rate of 0.6 mL/min at 55 °C. To compare stress tolerance properties of the *SPT15* mutant strains with the wild-type strain BY4741, PCA for fermentation data at each condition, including cell growth, glucose consumption and ethanol production at all the time points during fermentation (hours 0, 6, 12, 18, 24, 30, 36, 42 and 48). Packages FactoMineR and Factoextra [88] were used within R environment [89] for the PCA data analysis and ggplot2-based visualization, respectively.

### Transcriptome analysis

Six strains including three stress-tolerant mutants A140G, P169A and R238K, two stress-sensitive mutants S118L and L214V, as well as the wild-type strain BY4741 were subjected to transcriptome analysis using RNA sequencing. Flask fermentation of each strain were carried out in biological duplicates at stress and normal conditions, respectively. Cells were incubated to log phase, which required 9 h for normal conditions, 21 h for hyperosmotic stress conditions, 15 h for thermal stress conditions, and 33 h for ethanol stress conditions, respectively. Since cell growth was severely hampered at ethanol stress condition, qualified RNA failed to be extracted and used for the subsequent RNA sequencing. Cells were harvested in precooled Falcon tubes in liquid N_2_ by centrifuging for 5 min. The supernatant was removed, and the pellet was stored at − 80 °C until further use.

RNA extraction and sequencing libraries were prepared and sequenced on the Illumina platform using 150-bp paired-end sequencing by Hangzhou Lianchuan Biological Information Technology Co., Ltd. (Hangzhou, China). The sequencing data have been deposited in the Gene Expression Omnibus database (GEO, http://www.ncbi.nlm.nih.gov/geo/) under the accession number GSE160256. Differential gene expression (DEG) in comparisons of the *SPT15* mutant strains versus the wild-type strain BY4741 at each culture condition was analyzed as previously described [90]. Significantly differentially expressed genes (SDEGs) in each comparison were then extracted by applying an absolute fold-change threshold of 2.0 or greater and a false discovery rate (FDR)-corrected cutoff *p*-value of 0.05 or less (Additional file 5: Dataset S1, Additional file 6: Dataset S2, Additional file 7: Dataset S3), and further tested for Gene Ontology (GO) biological process enrichment using FunSpec with a *p*-value cutoff of 0.01 and Bonferroni correction [49] (Additional file 9: Dataset S5B). To identify transcription factors and significantly differentially expressed transcription factors (SDE-TFs) that may be responsible for regulating SDEGs, the SDEGs were searched against all of the transcription factors or SDE-TFs in the YEASTRACT database using the TFRank method in the tool of Rank by TF [61].

### Variant effect prediction and protein structure alignment

To look into functional effect of altered primary protein structure, the MutFunc database was employed to computationally predict the impact of protein sequence variants [57]. Pairwise protein structure alignment between Spt15 point mutation and the wild-type Spt15 was conducted using Schrodinger Maestro version 10.6 (Schrodinger, Inc., LLC, New York, USA) by following the instructions (Schrödinger Release 2020-3: Maestro, Schrödinger, LLC, New York, NY, 2020.). Two crystal structures of Spt15-related complexes (PDB 1YTB and 4B0A) were retrieved from protein data bank (PDB database, www.rcsb.org). 1YTB is a four-chain structure of the complex formed by the conserved carboxy-terminal domain of the yeast TATA-box binding protein (TBP) Spt15 and a 12 base pair (bp) duplex containing the TATA box of the *CYC1* promoter [73]. 4B0A is a structure of Spt15 with Taf1 N-terminal domains TAND1 and TAND2, showing competitive multiprotein TBP interplay patterns also presented by the structurally distinct TFIIA, Mot1 and Brf1 proteins and critical to transcriptional regulation [74]. The downloaded protein structure was prepared prior to docking using Schrodinger Maestro release 2020-3. Protein preparation was performed as previously reported [91]. Superposition of Spt15-Taf1 of the structure 4B0A onto the Spt15-DNA (Chain A and C) of the structure 1YTB was super-aligned using the PyMol program (http://www.pymol.org).

## Supplementary Information

**Additional file 1: Figure S1, S2.**

**Additional file 2: Table S1.** Predicted functional residues of Spt15 using the MutFunc database.

**Additional file 3: Table S2.** The resulting nucleotide and amino acid mutations in Spt15 using base editing.

**Additional file 4: Table S3.** Primers used in this study.

**Additional file 5: Dataset S1.** SDEG lists in the comparisons of Spt15 mutants vs. WT at normal condition.

**Additional file 6: Dataset S2.** SDEG lists in the comparisons of Spt15 mutants vs. WT at hyperosmotic stress condition.

**Additional file 7: Dataset S3.** SDEG lists in the comparisons of Spt15 mutants vs. WT at thermal stress condition.

**Additional file 8: Dataset S4.** GO Biological Process analysis of SDEGs.

**Additional file 9: Dataset S5.** Clustered TFs (A) and SDE-TFs (B) regulating SDEGs.

## Data Availability

All data generated or analyzed during this study are included in this published article and its additional files.
