# Supplementary material for: Stress tolerance enhancement via SPT15 base editing in Saccharomyces cerevisiae
Source: Biotechnol Biofuels. 2021 Jul 6;14:155. doi: 10.1186/s13068-021-02005-w (PMC8259078; doi:10.1186/s13068-021-02005-w)
Supplement: Supplementary file 1 — Additional file 1: Figure S1, S2. [file 13068_2021_2005_MOESM1_ESM.pdf]

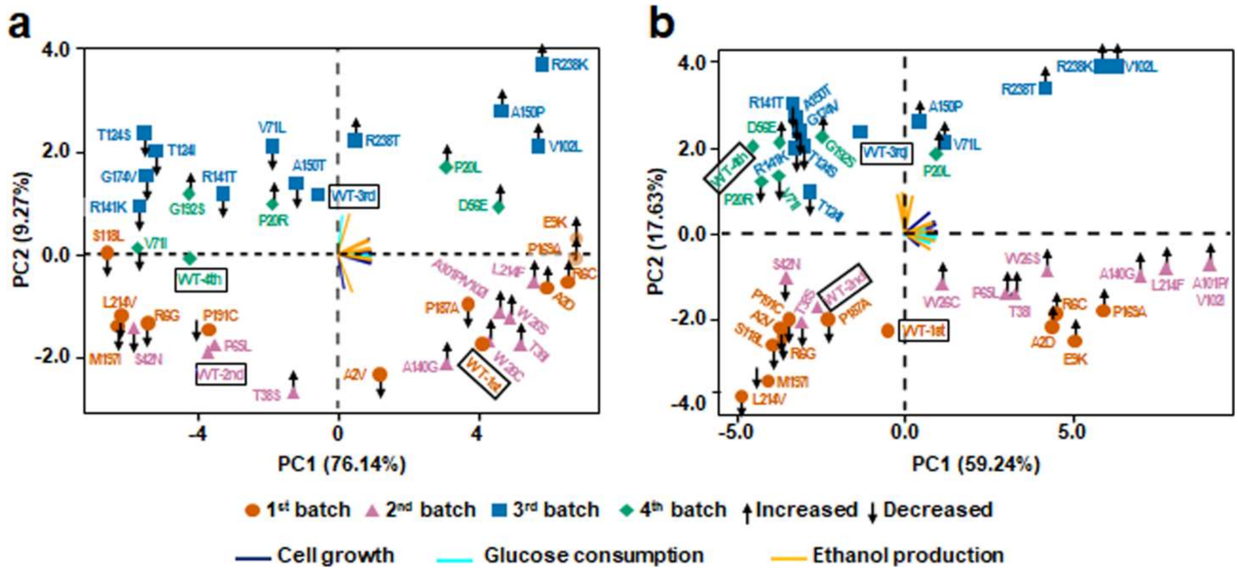

Fig. S1 Principal component analysis of fermentation data at thermal (a) and ethanol (b) stress conditions.

Fermentation data, including cell growth (purple lines), glucose consumption (turquoise lines) and ethanol production (orange lines) during fermentation (hours 0, 6, 12, 18, 24, 30, 36, 42 and 48), were from all the 36 Spt15 mutant strains and the wild type strain BY4741 at normal and three stress conditions. Means of biological duplicates are used. The strains were evaluated in 4 batches, indicated using the circle, triangle, square and diamond symbols, respectively. Compared to the wild type strain in each batch, mutant Spt15 strains showing increased or decreased fermentation capacities are indicated by upward arrow or downward arrow, respectively.

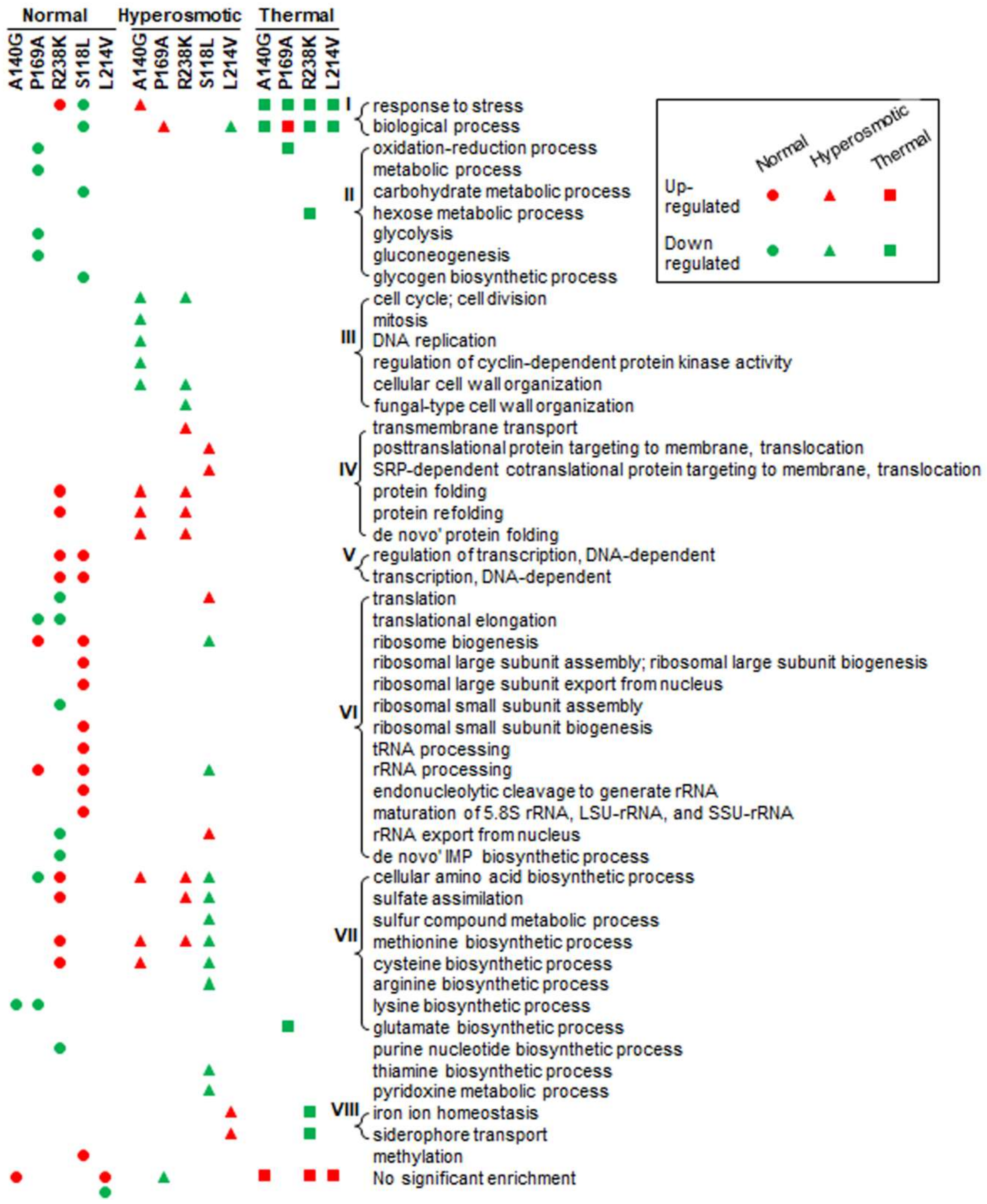

Fig. S2 Enriched biological processes of SDEGs influenced by key Spt15 mutants.

Significantly differentially expressed genes (SDEGs) were extracted in comparisons of the key Spt15 mutant strains versus the wild type strain BY4741 at each culture condition

including normal (circle symbol), hyperosmotic (triangle symbol) and thermal (square symbol) stress conditions. Up-regulated and down-regulated SDEGs were separately subjected to GO enrichment analysis, which are indicated in red and green, respectively.
